# Improving the efficiency and standards of a National Immunization Program Review: lessons learnt from United Republic of Tanzania

**DOI:** 10.11604/pamj.2017.28.209.10466

**Published:** 2017-11-07

**Authors:** Dafrossa Lyimo, Christopher Kamugisha, Emmanuel Yohana, Messeret Eshetu, Aaron Wallace, Kirsten Ward, Carsten Mantel, Karen Hennessey

**Affiliations:** 1 Ministry of Health & Social Welfare, Dar Es Salaam, Tanzania; 2 World Health Organization, Tanzania Country Office, Dar Es Salaam, Tanzania; 3 World Health Organization, Intercountry Support Office for Eastern and Southern Africa, Harare, Zimbabwe; 4 US Centers for Disease Control & Prevention, Atlanta, GA, USA; 5 World Health Organization, Headquarters, Geneva, Switzerland

**Keywords:** Immunization, evaluation, review

## Abstract

A National Immunization Program Review (NIP Review) is a comprehensive external assessment of the performance of a country’s immunization programme. The number of recommended special-topic NIP assessments, such as those for vaccine introduction or vaccine management, has increased. These assessments often have substantial overlap with NIP reviews, raising concern about duplication. Innovative technical and management approaches, including integrating several assessments into one, were applied in the United Republic of Tanzania’s 2015 NIP Review. These approaches and processes were documented and a post-Review survey and group discussion. The Tanzania Review found that integrating assessments so they can be conducted at one time was feasible and efficient. There are concrete approaches for successfully managing a Review that can be shared and practiced including having a well-planned desk review and nominating topic-leads. The use of tablets for data entry has the potential to improve Review data quality and timely analysis; however, careful team training is needed. A key area to improve was to better coordinate and link findings from the national-level and field teams.

## Introduction

National immunization program (NIP) Reviews are the core of a country’s monitoring and evaluation activities; they guide implementation of immunization services nationwide and help track progress towards goals of the national programme and of the Global Vaccine Action Plan 2016-2020 [1,2].

The World Health Organization (WHO) recommends that Member States conduct a comprehensive NIP Review every five years to guide the development of a new comprehensive multiyear plan for immunization (cMYP) [2]. NIP Review teams include global and local experts who collect, observe and synthesize program information across all levels (national to health facility) over a 2-3 week period.

The WHO Regions have developed Review guidelines [3,4]; however, reviews have grown increasingly complex, with concerns raised about variable quality across reviews. Additionally, increasing numbers of special-topic NIP assessments have been recommended, including post-vaccine introduction evaluations (PIE), vaccine-preventable disease surveillance reviews, vaccine supply management evaluations, data quality reviews, and further donor-specific evaluations [3-9]. These activities often have substantial overlap with NIP reviews, raising concern about duplication and NIP staff time spent conducting assessments [6].

Tanzania’s immunization program is one of the best performing in Africa with >90% of children completing three doses of Diphtheria-Tetanus-Pertussis-Hepatitis B- *Haemophilus influenza* type b (DTP-HepB-Hib) vaccine during 2010-2014. The country continues to strive to improve the program, especially to reach high coverage in all districts (12% of districts had <80% coverage with three doses of DTP-HepB-Hibvaccine in 2014) and to increase measles 2^nd^ dose coverage (29% in 2014). A NIP Review was planned for 2015 in preparation for developing the cMYP for 2016-2020.

This report outlines lessons learned from applying innovative strategies to the 2015 NIP Review in Tanzania.

## Methods

To improve the quality and efficiency of the NIP Review, Tanzania MoHSW and WHO Tanzania implemented innovative Review strategies and assessed their feasibility and acceptability. Five such strategies used were:

*Extensive integration of assessments:* this Review integrated the measles-rubella (MR) PIE, an assessment of the human papillomavirus (HPV) vaccine demonstration project, and assessments of data systems and quality, VPD surveillance and immunization financing.

*Modified desk review:* new timing and tasks of the desk review were tested. It is generally recommended [4] to conduct a desk review several months in advance and submit a report of findings. In this Review, the desk review was started 6 weeks before the field activities by a team of two people (one local staff and one external consultant) and was completed two weeks prior to field activities. Additional tasks were to immediately integrate desk review findings into tool development and Review team training.

*Selection of topic-leads:* this Review nominated topic-leads responsible for facilitating discussion and drafting conclusions and recommendations for their respective topic areas. Topic-leads were identified before field deployment by reviewing the expertise of all external Review members and making the best match of skill sets with topic areas.

Interactive review team training: the training included practical sessions on using hand-held tablets and mock interviews to familiarize the teams with the data collection tools and country context. A trainer initially guided each team through the data collection tools and use of the handheld tablets. Afterwards, each individuals within each team conducted mock interviews with one another to familiarize themselves with each question in the data collection tool and with use of the handheld tablets. Feedback was immediately collected from each team to identify any problems encountered with either the tablet or the data collection tool.

*Streamlined data collection tools and use of hand-held tablets:* as part of a regional effort to develop a standardized methodology for conducting Reviews in the African region, A team of experts from global agencies (WHO Headquarters, WHO African Regional Office, Centers for Disease Control and UNICEF) reviewed tools from previous reviews to identify core variables. The core variables were provided to the team Topic Leads conducting the Tanzania Review and these variables were enhanced based on the desk review findings and objectives of the other assessments being integrated. Topic leads reviewed the respective segment of the questionnaires to ensure desk review findings and local context were taken into account. Field teams used a tablet containing the data collection tools programmed with Zegeba software [10].

*Evaluation of review strategies:* the following methods were used to evaluate the review strategies.

*Post-review survey:* to assess the acceptability and feasibility of strategies, all reviewers were asked to complete a standard post-review evaluation form.

*Post-review group interview:* an interactive group session was held at the end of the Review to obtain feedback on process and practical experiences from the Review.

*Written feedback:* the review process was documented through reports written by the desk review consultant and the review-lead.

These evaluation findings are presented according to the five NIP Review phases: 1) concept development, 2) preparation, 3) implementation, 4) synthesis of conclusions and recommendations, and 5) translating recommendations to action [2].

## Results

The Tanzania Review was conducted from 8^th^ to 24^th^July 2015 and included 22 national and 10 international participants. The post-Review survey was completed by 22(66%) participants. Of these, over half (55%) had previously participated in at least one NIP Review. The post-Review interactive group session was attended by 25 persons, of whom over 90% participated as field team reviewers.

*Phase 1: concept development*

The concept note was developed six months prior to the review and was used to secure national participation and help partner agencies nominate external reviewers with the required expertise as topic leads.

*Phase 2: preparation*

An international consultant was hired for four weeks to conduct the desk review and assist with tool development and team training. A second international consultant was hired as the overall Review-lead, responsible for Review team training, oversight, debriefing and report writing. Additionally, eight external expert reviewers served as topic-leads on: NIP management and financing, vaccine management, service delivery, monitoring and data quality, surveillance, community demand, measles-rubella and HPV vaccine introduction.

The desk review summarized the status of recommendations from six large assessments during 2010-2014 including the 2010 NIP Review, three PIEs, a VPD surveillance review and a vaccine management assessment. Immunization stakeholders were interviewed to help assess status of recommendations from these previous assessments and identify priority areas for the Review.

*Phase 3: review implementation*

**Figure 1:**
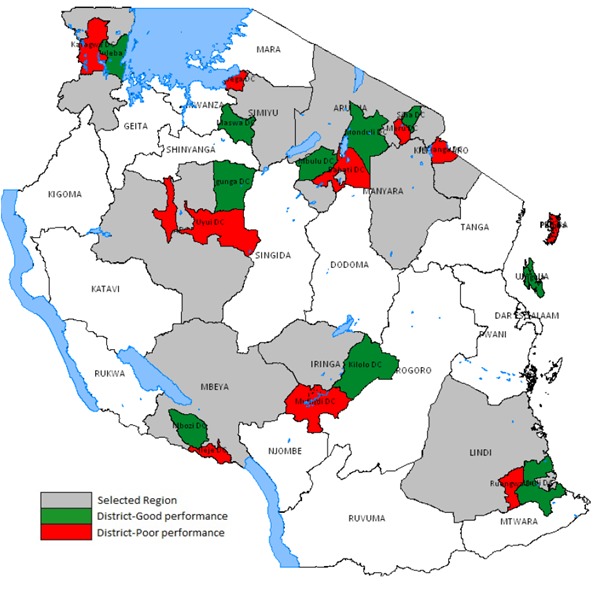
The 10 regions and 20 districts selected for field assessment in the 2015 National Immunization Program (NIP) Review in Tanzania

The NIP Review questionnaires were shortened by at least 30% (based on number of questions) compared to questionnaires used in other recent NIP Reviews. Ten of 30 regions in Tanzania were purposefully selected to be representative of the geographic variations in the country; within each region, one high-performing district and one low-performing district were randomly selected from a high-performing and low-performing sampling frame (performance based on DTP3 vaccination coverage) (Figure 1). Within each district, two health facilities were randomly selected. Ten teams each covered one region and in total visited 10 regional and 20 district health offices and 40 health facilities, observed 33 immunization sessions and 123 children being vaccinated, and interviewed 126 caregivers of these children (three children had two caregivers with them and both caregivers were included in interviews). Each team spent approximately five days within each region visiting their assigned locations for the Review. In addition, two international reviewers conducted the national-level aspect of the Review by having 21 key informant discussions with government officials and immunization partners.

Among the surveyed Review participants, 60% found integration of multiple assessments acceptable and 65% found the length of the tools acceptable (Table 1). Participants who did not find integration of multiple assessments acceptable expressed that it may only be feasible with good country capacity and support to do so. Participants who did not find tools acceptable felt that queries were missing in specific topic areas namely surveillance or new vaccine introduction. The tablets used for data collection were well accepted by 100% of the surveyed participants, with approximately half believing the tablets helped increase accuracy and decrease workload. Participants reported that tablet-based data were available in a timelier manner for analysis compared to paper-based tools.

**Table 1:** Results from a survey completed by 22 (66%) reviewers participating in a comprehensive national immunization program review, United Republic of Tanzania, July 2015

Area	Query	Percent in Agreement	Percent in Disagreement	Percent Unsure
Integrating multiple assessments	The number of objectives integrated in assessment was acceptable	60%	40%	0%
Data collection tools	The length of data collection tools was acceptable	65%	35%	0%
	Questions were acceptable	44%	56%	0%
	Balance of quantitative and qualitative questions was acceptable	62%	38%	0%
Use of Tablets	Tablet use was easy or acceptable	100%	0%	0%
	Tablets increased accuracy of data entry	47%	6%	47%
	Tablets decreased work load	47%	29%	24%
	Tablets disrupted flow of interviews	24%	76%	0%
	Recommend tablets for future reviews	82%	18%	0%
Training	Felt adequately briefed on country policies/strategies prior to field review	63%	37%	0%
Synthesis of Findings	Had sufficient time to synthesize field data and develop conclusions and recommendations	86%	14%	0%

*Phases 4 & 5: synthesis of conclusions and recommendations and translation into action*

Field teams reconvened and presented findings from the region they visited, while topic-leads consolidated the respective topic-area findings from all teams into a topic-area report and presentation. Immediately afterwards, topic-leads led breakout groups to discuss and draft cross-teamconclusions and recommendations. Back in plenary, topic-specific conclusions and recommendations were presented to ensure agreement. Linking findings from the national and field teams was limited due to intense focus on the field team findings. Of the surveyed reviewers, 86% reported having sufficient time to synthesize findings. The Review concluded with a presentation to MOHSW and key NIP partners; Review findings and recommendations that were endorsed by MOHSW and NIP partners were used to develop the 2016-2020 cMYP.

## Discussion

Tanzania was able to successfully implement a high quality integrated NIP Review. The findings and recommendations of the Review were summarized and translated into action plans during a subsequent workshop to develop the 2016-2020 cMYP. Although a number of these innovations may have been utilized on an ad-hoc basis in prior reviews, this Review is among the first to use a systematic approach to implementing multiple innovations together as a single package with the goal of streamlining the Review while also ensuring this Review provides sufficient information to answer questions that would normally be answered through conducting several separate reviews. Additionally, the Tanzania Review provided an opportunity to document lessons learned that were later incorporated into new integrated NIP review guidelines which are designed to ensure countries need only conduct a single activity to answer the many questions which originally had to be answered through the conduct of several separate activities. These lessons are summarized in Table 2.

**Table 2:** Summary of approaches and lessons for improving the quality and efficiency of the national immunization programme (NIP) review and lessons learnt, United Republic of Tanzania, July 2015

Activity	Challenge addressed	Approach taken	Lessons learnt
Phase 1: Concept note development & desk review	NIP reviews often are externally driven, can lack focus on country priorities	Concept note was used to secure national participation and input Desk Review identified priorities and barriers Tools focused on priorities	The concept note, desk review and tailored data collection tools helped engage the country program and shape the review to meet country needs.
Phase 1 &2: Integration of multiple assessments	NIPs must conduct multiple assessments with overlapping themes	The Review was designed to integrate multiple assessments	Reviewers found integrating assessments to be feasible; country NIP staff reported this to be highly desirable and efficient
Phase 2: Review management (Topic-leads)	Asingle Review-lead often does not have the breadth of technical knowledge needed to lead all topics of the review	Identified reviewers with relevant expertise to serve as topic-leads to synthesizeconclusions and recommendations (C&Rs)in their respective topic areas Drafted detailed responsibilities for Review-lead and topic-leads	Expert topic-leads can significantly improve the quality and relevance of conclusions and recommendations A Review-lead should be engaged at least 2 weeks before and after the Review for preparation and completion of final report
Phase 2: Developing data collection tools	Data collection tools are often too lengthy and lack focus on priorities	Tools cut by 30% from generic NIP data collection tools Desk review drove content Removed topics recently assessed	A stream-lined tool helped focus on priorities and extra time allowed teams to have more time to explore barriers and solutions
Phase 2: Team training	Review teams are often not well-briefed on country programme, status, data collection tools	The desk review guided the content of team training Included a practicum on tools and mock interviews as a way to familiarize reviewers with tools and the country programme	A 3-day training provided adequate time to prepare participants for field review Linking the desk review findings to the training to tighten the transfer of status of recommendations and barriers to the teams
Phase 3: Use of hand-held tablets	Field review data are often not ready in time to be used for debriefing session	Tablet was given to each team Valid entries and drop-down options were programmed Used tablet camera to verify findings with photos	Tablets allowed timely data analyses to support final presentation development Training is needed to prevent interviews that are focused on tablet data entry rather than discussion
Phase 4: Synthesis of conclusions and recommendations	Developing a reasonable number of meaningful and actionable recommendations can be challenging	Topic-leads facilitated discussion and drafted initial conclusions and recommendations National staff and partners actively participated in drafting recommendations	Topic-leads were important for leading discussion and generating meaningful recommendations Deliberate efforts are needed to integrate findings from the national-level review with the field review
Phase 5: Translate recommendations into action	Review recommendations may not lead to actionable work plans	The Review was intentionally scheduled to be completed some weeks before a workshop for developing the NIP’s new comprehensive multiyear plan (2016-2020)	Linking persons responsible for the NIP Review and the cMYP and timing both activities is critical for translating Review recommendations into NIP action plans.

The approach to conducting an integrated review as a means to efficiently address several different objectives simultaneously (i.e. introduction of a new vaccine, evaluation of vaccine supply chain management, general program evaluation etc) has gained traction in recent years, with examples of countries where the Review leads choose a more integrated approach, including in Ghana and Rwanda. In these latter two reviews, the general aim was to incorporate a traditional NIP review with a post-vaccine introduction evaluation (PIE) rather than conducting two separate activities. In contrast, for Tanzania, our regional team of immunization experts aimed to design this integrated review to address the needs of a traditional NIP review, PIE, financial records review, supply chain management review, and surveillance systems review. Additionally, the integrated review leads shared this aim through the review concept note with all immunization program stakeholders to ensure that their needs would be met by the single review and that they would not mandate an additional separate review. Additionally, we incorporated a framework of efficiency measures, including using topic leads to consolidate the field review findings by topic, and using electronic data collection to accelerate data management and analysis. Lastly, we documented the feedback of all review participants in regards to both the integrated design of the review and the success of the efficiency measures as a means to feed into a draft set of best practices for conducting integrated NIP reviews. This best practices document will be disseminated soon on the WHO website for all countries to incorporate into their future NIP reviews.

Here we document lessons for future NIP Reviews which may also be relevant for other nationwide program evaluations. First, adetailed NIP Review concept note developed six months prior to the field review helped secure both national and international participation. Second, timely engagement of an experienced desk review consultant helped tailor the Review to meet country needs. Third, the majority of surveyed participants found that integrating multiple assessments was feasible and NIP management found it highly desirable for efficiency reasons. Fourth, reviewers recommended tablet use for future reviews with a major benefit being that quantitative data were available in time to support conclusions and recommendations. However, a staff member with specialized technical knowledge was required to support tablet use. Lastly, identifying experienced topic-leads allowed for highly technical and focused discussions on various topics to take place concurrently during the field teams’ debriefing sessions. In future Reviews, linking of national and field findings may be improved by engaging topic-leads earlier in the process and having explicit guidance for them to synthesize the two levels of findings.

Limitations and challenges exist in the innovations used in the Tanzania Review. Although we used electronic data collection to great success per the feedback provided by participants, the ability of sharing data rapidly with all participants requires cellular or wireless network availability which could be limited in a number of rural settings globally. Additionally, a specialized skillset is required to manage and troubleshoot computing devices; this skillset may not be available in all reviews, which could hamper use of electronic data collection. Another challenge is ensuring that topic leads with sufficient applicable experience are recruited to a review; generally a key aspect of these reviews is inclusion of an external reviewer who is likely to provide a less-biased perspective on the performance of the program, but sufficiently experienced external participants may not always be available for all reviews.

## Conclusion

Many reviews have been conducted in the WHO African Region and it is common for reviewers to report that topics are not fully assessed even when the long version of the tools are used. This is the nature of a comprehensive evaluation covering a broad range of technical areas. The finding that the majority of participants found integration of multiple assessments and streamlined data collection tools acceptable was considered a favorable outcome, especially given that data collection queries were reduced by at least 30%. This approach was made successful by having a thorough desk review and training that provided the rationale for eliminating or expanding certain topics.

### What is known about this topic

- National Immunization Program (NIP) Reviews are routinely conducted in low and middle income countries;
- Multiple other types of immunization program reviews are also conducted on an annual basis and may heavily overlap with NIP Review objectives.

### What this study adds

- Aligning and conducting multiple assessments at one time is feasible with appropriate planning;
- Using the desk review findings to develop tailored data collection tools and training improves the relevance of the Review;
- Nominating Review members to be topic-leads based on their topic-specific expertise can significantly improve the quality and relevance of conclusions and recommendations.

## Competing interests

The authors declare no competing interests.
